# The correlation between brain structure, function, and cognitive changes in patients with active-stage ulcerative colitis

**DOI:** 10.3389/fnins.2025.1686273

**Published:** 2026-01-12

**Authors:** Weijie Fan, Wei Li, Si Zhang, Haiyu Zhang, Baobao Huang, Xia Xie, Li Wen, Dong Zhang

**Affiliations:** 1 Department of Radiology, The Second Affiliated Hospital of Army Medical University, Chongqing, China; 2 Department of Radiology, 987th Hospital of Joint Logistics Support Force of Chinese People’s Liberation Army, Baoji, Shaanxi, China; 3 Department of Medical Imaging, Chinese Traditional Medicine Hospital of Dadukou District, Chongqing, China; 4 Department of Gastroenterology, The Second Affiliated Hospital of Army Medical University, Chongqing, China

**Keywords:** cognitive impairment, fractional amplitude of low-frequency fluctuations, gut-brain axis, ulcerative colitis, voxel-based morphometry

## Abstract

**Background:**

Patients with active ulcerative colitis (UC) frequently exhibit emotional disturbances and cognitive deficits. However, the neurobiological basis of these manifestations remains poorly understood. This study investigates neurostructural and functional alterations in UC patients using multimodal MRI to identify potential neural correlates.

**Methods:**

We enrolled 45 active-stage UC patients and 48 healthy controls, all of whom underwent structural MRI, resting-state functional MRI (rs-fMRI), neurocognitive testing, and clinical assessments. Regional neural activity was evaluated using fractional amplitude of low-frequency fluctuations (fALFF), while gray matter volume (GMV) was analyzed to assess structural differences. Brain regions showing significant abnormalities were further examined for correlations with cognitive performance and clinical scale results.

**Results:**

Compared to the healthy control group, the UC patient group exhibited higher scores in PSQI, PSS, SAS, and SDS. Furthermore, the UC patient group displayed varying degrees of impairment in attention, working memory, and executive function. The GMV of the bilateral thalamus in UC patients decreased, while the fALFF values in bilateral posterior cingulate gyrus (PCG) and left lingual gyrus increased. Conversely, the fALFF values in multiple brain regions, including bilateral frontal lobes, the right temporal lobe, and the right inferior parietal lobule, were decreased. Multiple brain regions with reduced activity in the bilateral frontal lobes are closely related to emotions and executive control, while the increased activity in the bilateral PCG is strongly correlated with stress and anxiety. The reduction GMV in bilateral thalamic is associated with working memory and attention.

**Conclusion:**

Cognitive impairment and emotional abnormalities in UC are associated with the functional activity and structure of multiple brain regions, particularly in the bilateral frontal lobes, PCG and thalamus. These findings provide potential neuroimaging evidence for the activation of the gut-brain axis due to chronic inflammation, and that certain brain regions may be considered as key targets for predicting cognitive impairment for UC patients.

## 1 Introduction

Ulcerative colitis (UC) is a chronic inflammatory bowel disease (IBD) that primarily affects the colon and rectum, and its etiology remains unclear (Le Berre et al., 2023). The main clinical manifestations of the disease include recurrent abdominal pain, diarrhea, and rectal bleeding (Kaenkumchorn and Wahbeh, 2020). However, UC patients are often associated with numerous neuropsychiatric problems, including increased stress, abnormal emotions (anxiety or depression) (Yuan et al., 2021), and cognitive functional impairment (Sharma et al., 2021). These problems can lead to a significant decline in the quality of life for UC patients (Rozich et al., 2020). With the changes in the modern biomedical model, clinicians are increasingly focusing on the neurophysiological issues of UC patients (Lamb et al., 2019), more and more researchers are attempting to explore the underlying causes of these problems.

Due to the prolonged course of UC disease, long-term chronic inflammation makes patients more susceptible to anxiety and depression (Bisgaard et al., 2022). However, the intricate interactions between the gut and the brain, particularly the bidirectional effects of the gut-brain axis (GBA), may be one of the key reasons for the recurrent episodes of UC disease, emotional abnormalities, and cognitive impairments (Westfall et al., 2021). Mikockam’s review analyzed the prevalence rates of anxiety and depression in IBD patients, which reach as high as 19.1% and 21.2%, particularly during active stage of the disease (Mikocka-Walus et al., 2016). Another research also found that patients with moderate to severe UC frequently exhibit symptoms of anxiety and depression, which significantly affect their quality of life (Oh et al., 2025). To explore the causes of cognitive impairment or emotional changes in IBD patients, neuroimaging tests such as functional MRI or structure MRI are often used to analyze brain function or structure (Mciver et al., 2025). Wang et al.’s (2023) research indicates that there is an enhanced functional connectivity between the medial prefrontal cortex (mPFC) and the posterior cingulate cortex (PCC) in patients with IBD, which is associated with cortical GABA levels and the severity of depression. Research by Kornelsen et al. (2022) found structural and functional changes in the brains of patients with IBD. Alterations were observed both within and between networks in the default mode, cerebellum, sensorimotor, and visual networks, suggesting the presence of functional abnormalities in the GBA in IBD patients (Thomann et al., 2021). Our previous research found that the decline in the function of the bilateral hippocampus and parahippocampal region in patients with UC is closely related to working memory, while the abnormal functional connectivity of the cingulate gyrus is closely related to executive functions (Fan et al., 2019). However, the previous studies have not comprehensively analyzed the relationship between brain structure, function, and the emotional and cognitive changes in patients with UC. Further neuroimaging evidence needs to be explored.

This neuroimaging study investigates structural and functional brain alterations in patients with active-stage ulcerative colitis (UC) through resting-state fMRI analysis. We employed two complementary neuroimaging metrics: (1) fALFF (fractional amplitude of low-frequency fluctuations) to assess regional neuronal activity levels, and (2) GMV (gray matter volume) to evaluate structural integrity. By correlating these neuroimaging markers with standardized emotional scale scores and cognitive test performance across all participants, we systematically identify brain regions exhibiting pathological changes. These analyses ultimately seek to elucidate the neural substrates contributing to affective disturbances and cognitive dysfunction in UC.

## 2 Materials and methods

### 2.1 Participants

A total of 45 patients with active-stage UC were included in this study, which were diagnosed by the Department of Gastroenterology of the Second Affiliated Hospital of the Army Medical University. All included patients underwent gastrointestinal endoscopy within 2 weeks prior to their head MRI scan, and were evaluated by a gastroenterology specialist for Mayo score and Montreal classification, and the time of illness was recorded. The inclusion of the patient group was labeled as follows: (1) IBD patients aged 18–60 years, right-handed; (2) with an education level above primary school, (3) clinical colonoscopy confirmed as UC patients, and the disease is in the mild-moderate active-stage (Mayo score was between 3 and 10), (4) no use of steroid drugs in the past 6 months, (5) voluntarily signed informed consent and agreed to participate in this study. The exclusion criteria for the patient group were: (1) MRI reveals significant structural abnormalities, (2) with a history of intestinal surgery or other surgeries, (3) with endocrine, cardiovascular, hepatic and renal insufficiency and hyperthyroidism and other diseases that affect cognitive dysfunction, (4) presence of any contraindication to MRI scan, and (5) head motion parameter was greater than 2 mm or greater than 2° during brain MRI scan.

A total of 48 participants were recruited through advertising at the Second Affiliated Hospital of Army Medical University. The range of age and education level of healthy control were the same as patient group. The Healthy controls were right-handed and did not have any history of gastrointestinal symptoms and positive gastrointestinal endoscopy. In addition, the subject of HC group did not use steroids and psychotropic drugs that could cause cognitive changes. The exclusion criteria for the HC group refer to the UC patient group.

This study was approved by the Medical Ethics Committee of Second Affiliated Hospital of Army Medical University (2022-Research-199-01). All research content and methods were implemented in accordance with the approved research protocol and related policies. The investigator informed all participants of the purpose, content and significance of this study, and then obtained the consent of the subjects and signed a written informed consent form. All MRI data of the enrolled subjects have undergone de-identification to protect the privacy of the subjects.

### 2.2 Clinical scales and cognitive testing

All participants were assessed by relevant clinical scales, including the Pittsburgh sleep quality index (PSQI), the Perceived Stress Scales (PSS), the Self-Rating Anxiety Scale (SAS) and the Self-Rating Depress Scale (SDS). In addition, UC patients underwent the Inflammatory Bowel Disease Questionnaire (IBDQ) and Visual Analogue Scale (VAS) to assess their quality of life with UC disease and the level of disease-induced pain. The cognitive tests were conducted on the E-Prime 2.0 platform^1^, including attention tests, Stroop test and 2-back test. The attention test can obtain the subject’s attention network by three indicators: reaction time (RT) of Alerting effect, Orienting effect and Executive effect. The Stroop test can analyze the subject’s ability to perform color-font working tasks. The participants’ working memory can be assessed using the 2-back test. The above cognitive functions are evaluated by accuracy (ACC) and RT.

### 2.3 MRI data acquisition

All participants were scanned using a Philips Ingenia 3.0 Tesla MRI system equipped with a 32-channel head coil. The imaging protocol included both high-resolution T1-weighted anatomical scans and resting-state functional MRI acquisitions. During scanning, participants were instructed to remain awake with their eyes closed while maintaining a relaxed, motionless state. The parameters of T1 high-resolution MRI scan were as follow: repetition time (TR) = 7.0 ms, echo time (TE) = 3.2 ms, number of slices = 192, slice thickness = 1.0 mm, slice gap = 0 mm, voxel size = 1 mm × 1 mm × 1 mm, flip angle (FA) = 15°. The parameters of resting-state functional MRI scan were as follow: TR = 2,000 ms, TE = 30 ms, number of slices = 33, slice thickness = 3 mm, slice gap = 0 mm, voxel size = 3 mm × 3 mm × 3mm, FA = 90°, number of time points = 240.

### 2.4 MRI preprocessing and calculation

The preprocessing of resting-state functional MRI and T1 high-resolution MRI were performed by the corresponding software package on the MATLAB R2022b platform^2^. The extension package Computational Anatomy Toolbox 12 (CAT12^3^) in Statistical Parameter Mapping 12 (SPM12^4^) software was used for the preprocessing of T1 high-resolution MRI data, which provides Voxel-based morphometry (VBM) and Surface-based morphometry (SBM) analysis. The first step in VBM processing is the segmentation of brain tissue after the T1WI dicom to nifti conversion. Unified segmentation was performed according to the SPM’s standard by the spatial adaptive non-local means denoising filter. Then segmented white matter, gray matter, and cerebrospinal fluid were obtained, this step can help to optimize the subsequent automatic segmentation of the CAT. The second step in VBM processing is spatial normalization, which registers the segmented brain tissue of individuals into a standardized template for the Montreal Institute of Neurology (MNI) space. In the final step, an 8 mm full width at half-maximum (FWHM) Gaussian kernel is applied to smooth the modulated gray matter image for subsequent statistical analysis. In addition, extract the total intracranial volume (TIV) values of each individual to serve as covariates for further analysis after data quality control.

The pre-processing of resting-state functional MRI was conducted using the restplus v1.28 toolbox^5^ on the MATLAB R2022b, which included the following steps. After converting the Dicom image to nifti format, the first 10 time points were removed to avoid interference from uneven magnetic fields or noise. After that, slice-timing correction was used to synchronize the signals at different time points across the entire brain. Next, realignment and head motion correction were performed, and the head movement parameters were calculated using Friston-24 head motion correction. In the process, the subject with excessive head movement whose translations exceed 2.0 mm, and rotations exceed 2° need to be excluded. This step allows for the adjustment of the time difference between head movement and the slice. Co-register the above calibrated functional images to the corresponding T1 high-resolution structural images. The T1 structural image was segmented into white matter, gray matter, and cerebrospinal fluid for subsequent processing. The matched gray matter was spatially normalized by dartel to the standard MNI template for group comparison and resampled at 3 mm × 3 mm × 3 mm. Then, a gaussian kernel of 6 mm FWHM was used to spatially smooth the function data. Meanwhile, the fMRI data removed linear trends for detrending. In addition, potential influencing factors such as head motion parameter, global brain signals, white matter, and cerebrospinal fluid signals were removed as covariates to reduce noise. Finally, the processed functional data was filtered at the 0.01–0.08 Hz band.

Whole brain fALFF analysis was performed on preprocessed fMRI data using a 0.01–0.08 Hz bandpass filter to attenuate physiological noise from cardiac and respiratory cycles. The resulting fALFF values underwent Fisher’s r-to-z transformation to normalize the distribution for subsequent group-level comparisons. This analytical approach offers several advantages: As a normalized version of ALFF, fALFF minimizes contamination from high-frequency noise. The method provides enhanced sensitivity to physiologically relevant neural activity. The resulting signals better reflect genuine brain activity patterns by reducing non-neural fluctuations.

### 2.5 Statistical analysis

Statistical descriptive analysis of demographic, emotional scales, and cognitive function test results was performed using SPSS 26.0 software package^6^. For categorical variables such as gender, the Pearson’s chi-squared test was employed. A statistical analysis of continuous variables that follow normal distribution was conducted using a two-sample *t*-test. If the continuous variables were not normally distributed, we employed the Mann-Whitney rank test. All tests were conducted using two-tailed tests, with a significance level set at *p* < 0.05 to indicate statistically significant differences.

Based on the SPM12 software package, a two-sample *t*-test was used to detect the regional differences in gray matter volume (GMV) in CAT12 between the UC group and the HC group. The results were corrected by family-wise error (FWE) for multiple comparisons, with the voxel-level threshold at *p* = 0.001 and the FWE statistical significance of *p* < 0.05. In addition, gender, age, education, and TIV were used as covariates in this process. When analyzing the fALFF in the differences of brain functional activity between two groups, gender, age and education were considered as nuisance covariates. The multiple comparisons were corrected using FWE and an extent threshold of 30 voxels. Similarly, we set the voxel-level threshold to *p* = 0.001 and the FWE statistical significance of *p* < 0.05. Finally, correlation analysis was conducted using Pearson’s correlation to assess the relationship between the GMV or fALFF of the abnormal brain region, as well as clinical emotional or cognitive function parameters among two groups. The process was performed in SPSS 26.0 software, controlling for the effects of age, gender, and education, setting *p* < 0.05 as statistically significant with a two-tailed test.

## 3 Results

### 3.1 Demographic, clinical, and neuropsychological results

The results of the statistical analysis of demographics, clinical data, emotion and cognitive performance between UC patients and HC are shown in Table 1. There is no statistically significant difference in demographic between two groups, including age (*p* = 0.158), gender (*p* = 0.442) and education (*p* = 0.153). The sleep quality of the UC patient group was worse than that of the HC group, and their PSQI scores were higher (*p* = 0.042). In terms of emotional scales, the UC patient group obtained higher scores in PSS, SAS, and SDS compared to the HC group, with *p*-values of 0.033, 0.000, and 0.000, respectively. In the attention test, there were no statistically significant differences between the two groups in terms of alerting effect and orienting effect RT. However, the UC group exhibited a significantly longer RT in the executive effect compared to the HC (*p* = 0.029). Finally, the executive functional and working memory of the UC group are inferior to those of the HC group, as evidenced by the longer RT in Stroop (*p* = 0.002) and two-back tasks (*p* = 0.004) and the lower ACC in two-back task of the UC group (*p* = 0.041).

**TABLE 1:** Demographics, clinical data, emotion and cognitive performance among ulcerative colitis (UC) and healthy controls (HC) groups.

Characteristic	UC patients (*n* = 45)	Healthy controls (*n* = 48)	Statistic (*t*/χ^2^/*z*)	*P*-value
Age (years)	41.36 ± 14.01	37.31 ± 13.37	1.424	0.158^a^
Gender (male/female)	27/18	25/23	0.591	0.442^b^
Education (years)	12.00 (9.00–13.50)	12.00 (9.00–15.75)	−1.429	0.153^c^
Disease duration (months)	16.00 (4.50–49.00)			
Montreal classification	E1: E2: E3 = 9: 19: 17			
Mayo	6.07 ± 2.33			
IBDQ	152.20 ± 38.19			
VAS	1 (0–3)			
PSQI	5.00 (3.00–9.00)	4.00 (2.00–6.00)	2.035	0.042^c^
**Emotion**				
PSS	33.91 ± 12.08	28.96 ± 9.96	2.163	0.033^a^
SAS	42.04 ± 8.68	34.96 ± 9.41	3.766	0.000^a^
SDS	47.49 ± 9.84	36.85 ± 11.46	4.786	0.000^a^
**Attention**				
Alerting effect (ms)	47.33 ± 27.27	51.36 ± 24.83	−0.746	0.457^a^
Orienting effect (ms)	32.76 ± 21.85	28.36 ± 20.68	0.996	0.322^a^
Executive effect (ms)	107.63 ± 34.15	92.78 ± 30.56	2.212	0.029^a^
**Executive function**				
ACC of Stroop (%)	89.0 (76.0–94.0)	90.0 (82.5–94.0)	0.358	0.720^c^
Stroop RT (ms)	829.12 ± 156.28	737.52 ± 126.14	3.120	0.002^a^
**Work memory**				
ACC of two-back (%)	70.62 ± 13.86	76.77 ± 14.68	−2.074	0.041^a^
Two-back RT (ms)	700.36 ± 122.30	637.67 ± 79.71	2.946	0.004^a^

IBDQ, inflammatory bowel disease questionnaire; VAS, visual analogue scale; PSQI, pittsburgh sleep quality index; PSS, perceived stress scale; SAS, self-rating anxiety scale; SDS, self-rating anxiety scale; ANT, attention network task; ACC, accuracy rating; RT, reaction time.

^a^Two-independent-samples *t*-test.

^b^Chi-square test.

^c^Mann-Whitney rank test.

### 3.2 Brain structural results

**FIGURE 1:**
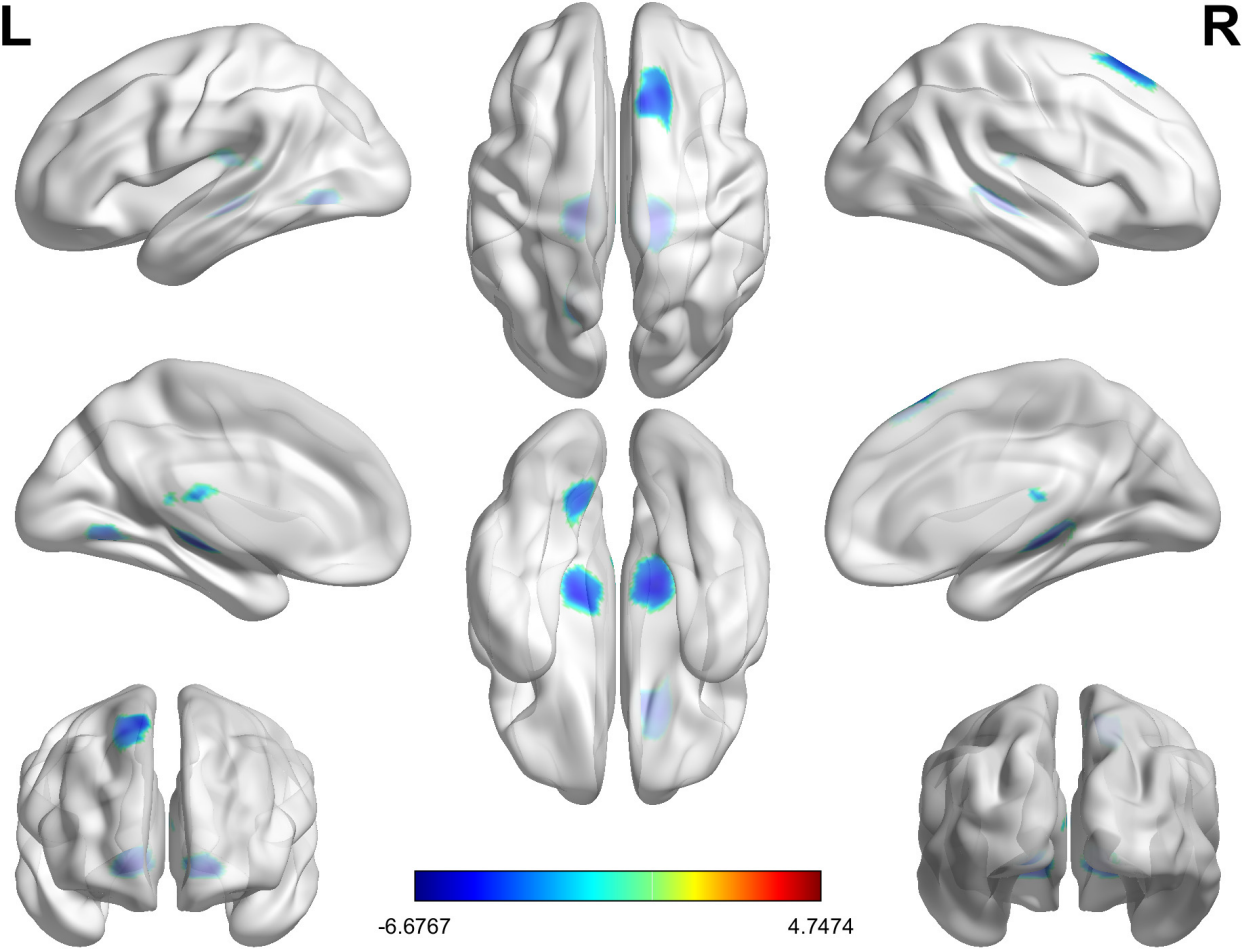
Changes in gray matter volume (GMV) values of the ulcerative colitis (UC) group relative to the HC group. The UC patient group exhibited significant reduced GMV value in the bilateral thalamus, right superior frontal gyrus and middle frontal gyrus, and left lingual gyrus compared to the HC group. The color bar represents the *T*-value of the two-sample *t*-test between two groups.

Details of regional differences in GMV between UC and HC groups were shown in Table 2 and Figure 1. The experimental results indicate that the GMV in three brain regions is diminished in the UC group compared to the HC group. The brain region with the largest decreased GMV of UC group is the bilateral thalamus, with a voxel size of 4,499 (*T* = −6.68). The GMV in the right superior frontal gyrus (SFG) and middle frontal gyrus (MFG) was the second decreased brain region, which has a voxel size of 1666 (*T* = −4.92). Lastly, the left lingual gyrus (LING) has a voxel size of 946 (*T* = −4.48) whose GMV decreased in UC group. All the three brain regions with abnormal GMV have been corrected by FWE (cluster-level threshold *p* < 0.05 and voxel-level threshold *p* < 0.001).

**TABLE 2:** Regional differences in gray matter volume (GMV) in ulcerative colitis (UC) group compared to the healthy controls (HC) group.

Brain region	Hem	Peak MNI coordinate			*T*-value	*P*-value	Voxel size
		X	Y	Z			
THA	L&R	−20	−15	−2	−6.68	0.000	4,499
SFG & MFG	R	10	24	56	−4.92	0.001	1,666
LING	L	−14	−62	−6	−4.48	0.021	9,46

Hem, hemisphere; MNI, Montreal Neurological Institute; THA, thalamus; SFG, superior frontal gyrus; MFG, middle frontal gyrus; LING, lingual gyrus; FWE correction (cluster-level threshold *p* < 0.05, voxel-level threshold *p* < 0.001).

### 3.3 fALFF analysis

**FIGURE 2:**
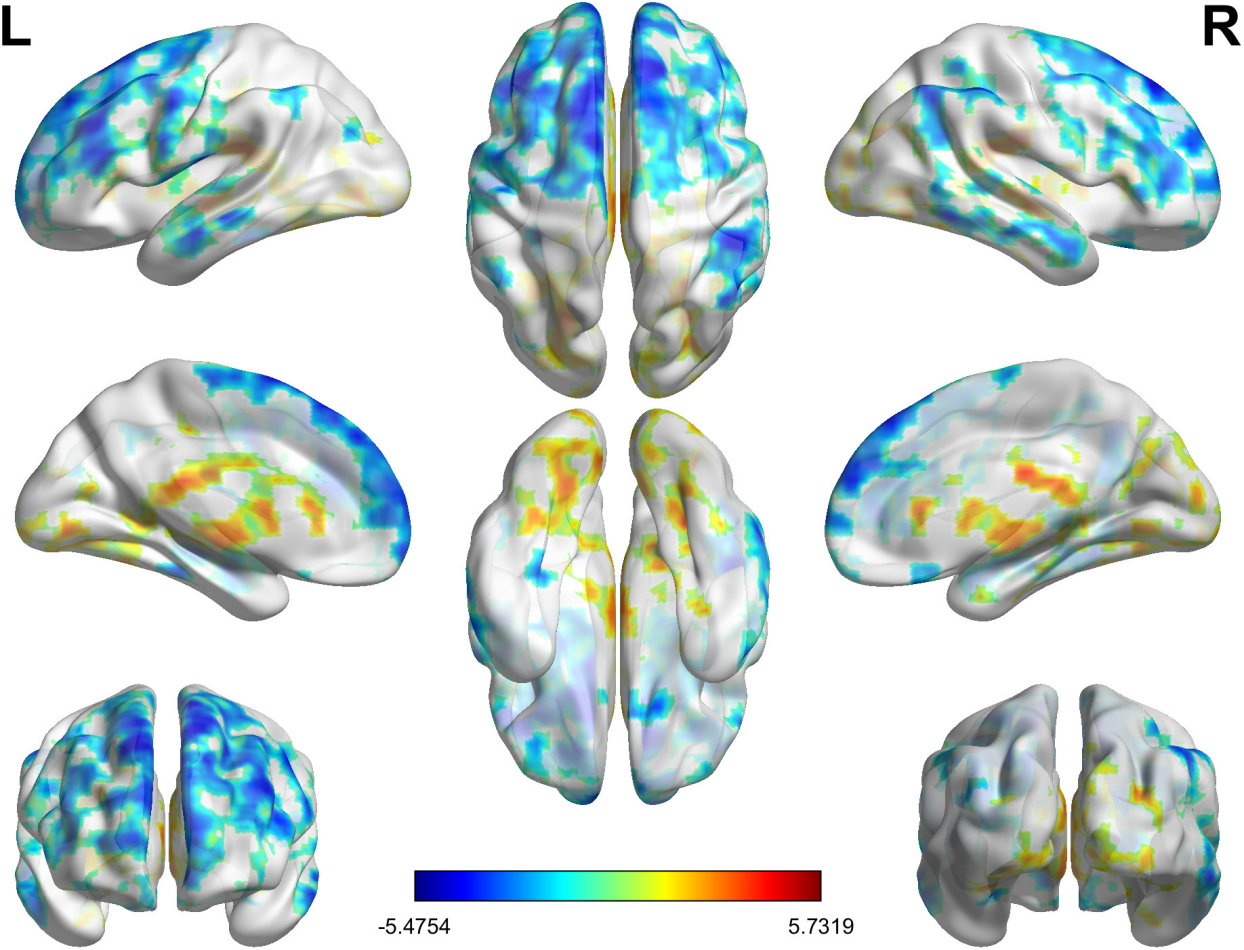
Changes in fractional amplitude of low-frequency fluctuations (fALFF) values of the ulcerative colitis (UC) group relative to the HC group. The significant increased (red) fALFF value in UC group compared with HC group include bilateral posterior cingulate gyrus, right calcarine fissure and left lingual gyrus. Conversely, brain regions with decreased (blue) fALFF values in the UC group included left superior frontal gyrus, right superior frontal gyrus and middle frontal gyrus, left middle frontal gyrus, left inferior frontal gyrus, right inferior parietal lobule, right middle temporal gyrus, and right inferior temporal gyrus. The color bar represents the *T*-value of the two-sample *t*-test between two groups.

Compared with the HC group, a total of 11 brain regions with abnormal fALFF values were found in the UC patient group which corrected by FWE with cluster-level threshold *p* < 0.05 and voxel-level threshold *p* < 0.001 (shown in Table 3 and Figure 2).

**TABLE 3:** Brain regions of abnormal fractional amplitude of low-frequency fluctuations (fALFF) value in the ulcerative colitis (UC) group compared to the healthy controls (HC) group.

Brain region	Hem	Peak MNI coordinate			*T*-value	*P*-value	Cluster size
		X	Y	Z			
PCG	R	15	−36	30	5.73	0.000	237
PCG	L	−24	−42	33	5.52	0.000	83
CAL	R	33	−69	15	4.89	0.030	34
LING	L	−21	−105	−9	4.23	0.045	31
SFG	L	−9	6	63	−5.28	0.000	167
SFG & MFG	R	15	36	45	−5.21	0.000	127
MFG	L	−39	33	27	−4.54	0.000	103
IFG	L	−60	6	33	−4.92	0.000	95
IPL	R	51	−36	48	−5.48	0.000	75
MTG	R	57	6	−27	−4.28	0.007	45
ITG	R	60	−48	−9	−4.69	0.018	38

Hem, hemisphere, MNI, Montreal Neurological Institute, PCG, posterior cingulate gyrus, CAL, calcarine fissure, LING, lingual gyrus, SFG, superior frontal gyrus, MFG, middle frontal gyrus, IFG, inferior frontal gyrus, IPL, inferior parietal lobule, MTG, middle temporal gyrus, ITG, inferior temporal gyrus, FWE correction (cluster-level threshold *p* < 0.05, voxel-level threshold *p* < 0.001).

Specifically, there are four abnormal brain regions with increased fALFF in the UC patient group, which include the left and right posterior cingulate gyrus (PCG), the right calcarine sulcus (CAL), and the left LING, with cluster sizes of 237, 83, 34, and 31, respectively. Additionally, the UC group exhibited a significant decrease in the fALFF values across seven clusters of brain regions, namely the left SFG, right SFG and MFG, left inferior frontal gyrus (IFG), right inferior parietal lobe (IPL), right middle temporal gyrus (MTG), and right inferior temporal gyrus (ITG).

### 3.4 Correlation analysis

**FIGURE 3:**
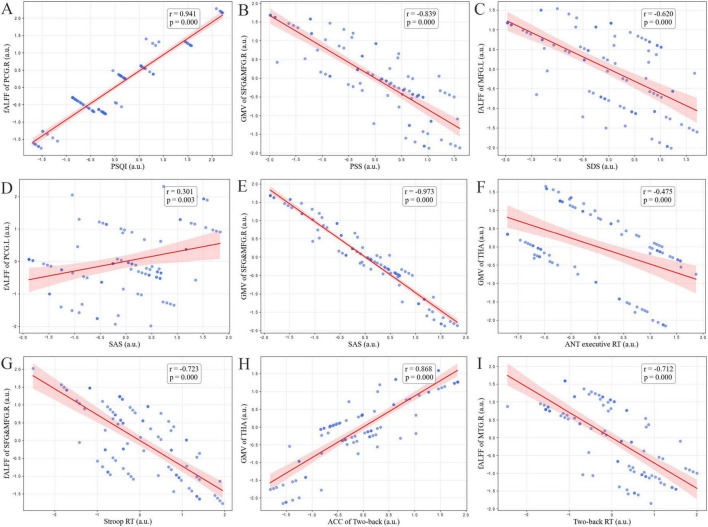
Correlations between abnormal fractional amplitude of low-frequency fluctuations (fALFF), gray matter volume (GMV) and neuropsychological assessment among all subjects. **(A)** The Pittsburgh sleep quality index (PSQI) score was positively correlated with the fALFF value of PCG.L. **(B)** The Perceived Stress Scales (PSS) score was negatively correlated with the GMV of SFG&MFG.R. **(C)** The Self-Rating Depress Scale (SDS) score was negatively correlated with the fALFF of MFG.R. **(D,E)** The Self-Rating Anxiety Scale (SAS) score was positively correlated with the fALFF value of PCG.L and negatively correlated with the GMV of SFG&MFG.R. **(F)** The ANT executive reaction time (RT) was negatively correlated with the GMV of THA. **(G)** The RT of Stroop was negatively correlated with the fALFF of SFG&MFG.R. **(H)** The accuracy (ACC) of Two-back was positively correlated with the GMV value of THA. **(I)** The RT of Two-back was negatively correlated with the fALFF of MTG.R.

The significant pairwise correlations between abnormal fALFF or GMV of brain regions, clinical scale score, and neuropsychological assessment among all subjects are illustrated in Figure 3. All data excluded the effects of age, gender, and education as covariates. We observed multiple positive and negative strong correlations as follows. First, the PSQI score was positively correlated with the fALFF value of PCG.L (*r* = 0.941, *p* = 0.000). Moreover, the PSS score was negatively correlated with the GMV of SFG&MFG.R (*r* = −0.839, *p* = 0.000). Furthermore, the SDS score was negatively correlated with the fALFF of MFG.R (*r* = −0.620, *p* = 0.000). Specifically, the SAS score was positively correlated with the fALFF value of PCG.L (*r* = 0.301, *p* = 0.003) and negatively correlated with the GMV of SFG&MFG.R (*r* = -0.973, *p* = 0.000). In addition, the ANT executive RT was negatively correlated with the GMV of THA (*r* = −0.475, *p* = 0.000). The RT of Stroop was negatively correlated with the fALFF of SFG&MFG.R (*r* = −0.723, *p* = 0.000). What’s more, in terms of working memory, we found that the ACC of Two-back was positively correlated with the GMV value of THA (*r* = 0.868, *p* = 0.000), and the RT of Two-back was negatively correlated with the fALFF of MTG.R (*r* = −0.712, *p* = 0.000).

## 4 Discussion

This study employed resting-state functional MRI and structural MRI to conduct a cross-sectional analysis of the gray matter structure and brain function in UC patients with active-stage and health controls. In terms of gray matter structure, the results indicated that the UC patient group exhibited a reduction in GMV value of the bilateral thalamus, the right superior and middle frontal gyrus, and the left lingual gyrus. In terms of brain function, the UC patient group exhibited multiple regions of functional impairment in the bilateral frontal gyrus, right temporal gyrus, and right inferior parietal lobule. These regions showed a strong correlation with the emotions of the subjects, particularly anxiety and depression. Conversely, the UC patient group showed increased activity in the bilateral posterior cingulate gyrus, the left lingual gyrus, and the right calcarine fissure, with most of these regions located in the limbic system. Correlation analyses also suggested significant ory. The findings of this study confirm alterations in the brain structure and fuassociations with cognitive functions, including executive function and working memnction of UC patients in active stage, indicating possible neurophysiological mechanisms underlying emotional and cognitive impairments in these patients, and providing relevant neuroimaging evidence.

It is well-known that the frontal lobe is a highly important functional region of the brain, including emotional regulation and multiple cognitive functions such as executive control (Hiser and Koenigs, 2018; Rolls, 2023). In this study, we found that the functional activity in some brain regions of the bilateral frontal gyrus of UC patients is diminished, and the GMV in the right SFG & MFG is reduced. These findings are consistent with several previous functional MRI studies and researches on DSS animal models of UC. Takahashi’s et al. (2023) research found that DSS mice exhibit abnormal myelination in the prefrontal cortex, and that brexpiprazole can prevent the occurrence of depressive-like behaviors by inhibiting the abnormal myelination. Research by Li et al. (2024) conducted a Mendelian randomization analysis to investigate the alterations in brain structural in IBD, found that GMV in the right superior frontal cortex was closely related to inflammation and may be involved in anxiety and depression. Moreover, our study also found that the PSS score of UC patients is negatively correlated with the GMV of the right SFG &MFG, which indicates that the increased perceived stress in UC patients may be related to the reduction in the volume of gray matter in the frontal lobe. Research indicates that adult rats with early life stress exhibit greater susceptibility to damage in the prefrontal cortex (Bercum et al., 2023). Another study demonstrated that chronic psychological stress was also a significant factor contributing to the exacerbation or recurrence of IBD (Ge et al., 2022). In conjunction with our research, the increase in stress among UC patients may lead to a GMV reduction in the frontal lobe, subsequently resulting in decreased activity across multiple brain regions in the frontal lobe, ultimately leading to anxiety and depression in UC patients.

Furthermore, our research found that SDS was negatively correlated with the fALFF value of the left MFG, and SAS was negatively correlated with the GMV of the right SFG & MFG in all subjects. Emotional control is one of the most important functions of the frontal lobe, and many studies have confirmed abnormal activities and structures of the frontal lobe in anxiety and depression (Roberts and Mulvihill, 2024). Mu’s et al. (2025) research found that altered structure-function synchrony in the frontal, temporal lobes, and THA may be implicated in the development of symptoms of anhedonia in depressive disorder patients. Dysfunction of the prefrontal cortex and anterior cingulate cortex is a characteristic of various anxiety disorders, with the circuitry of the prefrontal cortex playing a significant role in anxiety and depressive-like behaviors (Hare and Duman, 2020). Moreover, correlation analysis showed that the decrease in fALFF of right SFG&MFG was negatively correlated with Stroop RT in this study, suggesting that the right frontal lobe may lead to a decline in executive function. Executive function is a higher-order cognitive ability, and its main control brain region is the frontal lobe (Cristofori et al., 2019). Craine’s et al. (2023) study observed that rats with traumatic brain injury to the frontal lobe exhibited executive function deficits, which were alleviated following the improvement of frontal lobe function through the administration of Milnacipran. Alkan’s et al. (2024) research found that the GMV of the right MFG in patients with schizophrenia is correlated with performance on the Stroop test. Our research also found that structural and functional abnormalities in the bilateral frontal lobes of UC patients with active stage are closely associated with emotional disturbances and executive function impairments.

The thalamus, as the largest gray matter nucleus of the diencephalon, serves as an advanced center for sensation (Pan et al., 2023), while also participating in various cognitive functions, including sleep, working memory, and attention (Sweeney-Reed et al., 2021). Zhang’s et al. (2022) research found that patients with UC exhibit varying degrees of GMV reduction in brain regions such as the thalamus and insula, while another study found that changes in thalamic volume are closely correlated with C-reactive protein (Goodyear et al., 2023). Patel et al. (2024) also discovered a reduction in the GMV of the bilateral thalamus and hippocampus by investigating brain structures of 84 patients with IBD, and correlation analysis indicated an association with vascular comorbidities and a decline in working memory. It is well-known that the hippocampus plays a key role in the formation of memory, while the thalamus has an encoding function in the circuit that transmits hippocampal memory to the cortex (Toader et al., 2023). This role has been confirmed by multiple animal experiments (Dolleman-Van Der Weel et al., 2019; Morvan et al., 2022). This study also found a reduction in the GMV of the bilateral THA in UC patients, which negatively correlated with the RT of attention execution effect and positively correlated with the accuracy of the Two-back task. This suggests that the reduction in THA may lead to a decline in memory and attention in UC patients, and it may be related to the abnormal activation of the GBA.

The cingulate cortex plays a crucial role as an important brain component of the limbic system in the activation of the GBA (Aziz et al., 2021; Wu et al., 2023). Most studies regarding the brain structure or function in IBD have found evidence that colonic inflammation leads to changes in the structure and function of the cingulate cortex (Amlashi et al., 2025; Kornelsen et al., 2023). This study found that increased activity in the right PCG is positively correlated with the PSQI scores, while the fALFF of the left PCG is positively correlated with the SAS scores. These may demonstrate the potential mechanisms of the PCG in relation to sleep or anxiety in patients with UC. Li’s et al. (2023) research discovered the relationship between Forebrain-Posterior Cingulate and stress anxiety in patients with sleep deficiencies, and the functional reduction of the PCG and the basal nucleus is closely related to the PDSQ. Another study found multiple alterations in functional connectivity of the cingulate gyrus in chronic insomnia and anxiety symptoms, with increased functional connectivity with the thalamus and the MTG being the primary characteristics (Zhang et al., 2024). The structural and functional abnormalities of the bilateral THA and PCG may be important brain regions involved in the GBA, which interact bidirectionally with intestinal inflammation and affect cognitive functions such as attention and memory in UC patients.

In this study, the fALFF of right MTG showed a negative correlation with the RT of Two-back task in all subjects. Although the hippocampus is recognized as a core brain region for memory, numerous studies have confirmed that multiple brain regions in frontal and temporal lobes are also involved in the executive control processes of working memory (Christophel et al., 2024). Ohnishi et al. (2025) utilized voxel-based analysis to assess the brain structure of 57 patients with Alzheimer disease, founding that abnormal T1W/T2W ratio of the right MGT is closely associated with spatial cognitive dysfunction in AD patients and is involved in the formation of spatial memory. Moreover, Lee’s et al. (2022) study discovered that the frontoparietal network and the MTG can coordinate cognitive functions to control the formation of working memory using electroencephalography record. In addition, lingual gyrus and cuneus were responsible for both basic and higher visual processing (Palejwala et al., 2021). However, some researchers also believe that the lingual gyrus may be involved in visual memory (Yan et al., 2023), emotions and cognitive functions (Couvy-Duchesne et al., 2018; Liu et al., 2017). The findings in our study revealed a decrease in the GMV of lift Lingual gyrus in UC patients, along with an increased fALFF value. We speculate that this may be caused by some form of compensatory mechanism. However, the underlying mechanisms that UC patients have structural and functional abnormalities in the lingual gyrus in this study require further investigation.

This study still has several current limitations as follows. First, this study only compared the differences in brain function and structure between patients with active-stage UC and HCs, without conducting a follow-up analysis on the neuroimaging changes between the active and remission stage of UC. Second, potential influencing factors still require further analysis, such as the impact of different Montreal classifications of UC on brain function and structure. Third, the association and mechanisms between abnormal functional brain regions and structural changes in UC patients still need to be further explored. Finally, some patients with ulcerative colitis lacked recent systemic inflammatory markers, such as CRP, ESR, and fecal calprotectin, at the time of enrollment, which resulted in this study not analyzing laboratory indicators of inflammation. To address these limitations, future research should focus on the follow-up of UC patients and the analysis of changes in brain function and structure between different subgroups of Montreal classifications. Additionally, to better explore the potential physiological mechanisms of UC disease on brain structure and function, more animal experiments need to be designed.

## 5 Conclusion

This study explores the impact of UC disease on the brain structure and function of patients, identifying relevant brain regions that may lead to emotional changes and cognitive impairments in UC patients. The limbic system, as a crucial component of the brain-gut axis, plays a significant role in the progression of UC, wherein the reduced GMV in the bilateral thalamus and the increased functional activity in the bilateral PCG may be important brain regions contributing to various cognitive impairments in UC patients. The bilateral frontal lobes, being the most vital brain regions for emotional regulation in humans, show decreased functionality and atrophied GMV in multiple brain regions among UC patients in this study, suggesting a significant neural mechanism behind the patients’ emotional changes and cognitive impairment. These findings provide new insights for the diagnosis and treatment of UC, potentially offering meaningful implications for the prevention of neurocognitive impairments and improving patient prognosis in future UC management.

## Data Availability

The raw data supporting the conclusions of this article will be made available by the authors, without undue reservation.

## Footnotes

1
http://www.pstnet.com/eprime.cfm

2
https://www.mathworks.com/products/matlab.html

3
https://neuro-jena.github.io/cat12-help/#version

4
https://www.fil.ion.ucl.ac.uk/spm/software

5
http://www.restfmri.net/forum/RESTplus

6
https://www.ibm.com/products/spss
